# Remarkable change in age-specific breast cancer incidence in the Swiss canton of Geneva and its possible relation with the use of hormone replacement therapy

**DOI:** 10.1186/1471-2407-6-78

**Published:** 2006-03-22

**Authors:** Christine Bouchardy, Alfredo Morabia, Helena M Verkooijen, Gérald Fioretta, Yves Wespi, Peter Schäfer

**Affiliations:** 1 Geneva Cancer Registry, Institute for Social and Preventive Medicine, Geneva University, 55 boulevard de la Cluse, 1205 Geneva, Switzerland; 2 Division of Clinical Epidemiology, Geneva University Hospitals, 24 rue Micheli-du-Crest, 1211 Geneva 14, Switzerland; 3 Group of Gynecologists and Obstetricians Geneva, Association of Physicians of the Canton of Geneva, 8, rue Saint-Leger, 1205 Geneva, Switzerland; 4 Clinic of Gynecology, Senology, Department of Obstetrics and Gynecology, Geneva University Hospitals, 30 boulevard de la Cluse, 1211 Geneva 14, Switzerland

## Abstract

**Background:**

This article aims to explain the reasons for the remarkable change in age of breast cancer occurrence in the Swiss canton of Geneva.

**Methods:**

We used population-based data from the Geneva cancer registry, which collects information on method of detection, stage and tumour characteristics since 1975. For patients diagnosed between 1997–2003, we obtained additional information on use of hormone replacement therapy from a large prospective study on breast cancer. Using generalized log linear regression analysis, we compared age-specific incidence rates with respect to period, stage, oestrogen receptor status, method of detection and use of hormone replacement therapy.

**Results:**

In the periods 1975–1979 and 1985–1989, breast cancer risk increased with age, showing the highest incidence rates among women aged ≥ 85 years. From 1997, the age-specific incidence curve changed completely (p < 0.0001), showing an incidence peak at 60–64 years and a reduced incidence among elderly women. This incidence peak concerned mainly early stage and oestrogen positive cancers and was exclusively observed among women who ever used hormone replacement therapy, regardless whether the tumour was screen-detected or not.

**Conclusion:**

The increasing prevalence of hormone replacement therapy use during the 1990s could explain the important change in age-specific breast cancer incidence, not only by increasing breast cancer risk, but also by revealing breast cancer at an earlier age.

## Background

In Western countries, breast cancer is the most frequent cancer among women and its incidence is still increasing. Several factors have contributed to this increase. Screening mammography temporarily increases incidence by discovering incident, as well as prevalent cases. Higher exposure to breast cancer risk factors, such as low and late parity, alcohol intake and use of hormone replacement therapy (HRT) [1,2] could also explain the incidence increase. It has now been established that HRT represents a true risk factor for breast cancer [3,4]. After the publication of the Women's Health Initiative in 2002, a study reporting greater harm than benefit of HRT use [5], HRT use has decreased sharply in Europe and North America [6,7].

The Swiss canton of Geneva has one of the highest incidence rates of breast cancer in the world [8]. Opportunistic mammography screening was started in Geneva at the end of the 1980's and in 1999 an organised mammography screening program was implemented. This program invites all resident women aged from 50 to 69 years every two years to a free mammography with double reading. However, the participation in the organised screening program is low and most women undergo opportunistic screening. The Swiss national Health Survey, performed in 1997, reported that 66% of the Geneva women aged ≥40 years had undergone at least one mammography examination [9].

Use of HRT was very common in Geneva where over 50% of women aged 45–59 years used HRT in 1996 [10].

In this study, we describe changes in age-specific breast cancer incidence in Geneva and examine possible reasons for these changes.

## Methods

We used data from the Geneva cancer registry, which records information on all incident cancer cases that occur in the canton (approximately 420,000 inhabitants). The registration is based on several sources of information and is very accurate, as attested by its low percentage (<2%) of cases recorded from death certificates only [8]. All hospitals and pathology laboratories report information on every current and past cancer case to the Geneva cancer registry. Trained tumour registrars systematically abstract missing information and the cancer registry regularly sends special questionnaires to private physicians to secure information on patients treated in the private sector.

For every cancer case, we record sociodemographic characteristics, method of detection of the tumour, tumour characteristics, stage of disease at diagnosis, treatment, survival status, and cause of death.

The study included all 8045 invasive breast cancer cases diagnosed between 1975–2003 among the resident population. Method of detection was categorized as mammography screening vs. other. Stage at diagnosis was based on the Tumour, Node and Metastasis TNM classification system [11]. Stages were classified into three groups: stage I (T0 or T1, N0), stage II (T0 or T1 and N1, T2 and N0 or N1, T3 and N0), and other stages (T0 or T1 or T2 and N2, T3 and N1 or N2, T4 and any N, any T and N3, or M1, and unknown stages). Oestrogen receptor status was categorized as positive if tumours presented oestrogen receptors in ≥ 10% of the tumour cells.

Additional information on the patient's use of hormone replacement therapy (coded as ever vs. never user) was provided by a large study on risk factors for breast cancer [12]. This study was prospectively collected, by means of personal interviews, information on life style factors of breast cancer patients and control individuals.

This study included 400 breast cancer patients aged 50–74 years, diagnosed between 1997–2000 in the canton of Geneva and they were individually linked to the cancer registry data set. These women represented 46% of all breast cancer patients in this age group recorded at the cancer registry during the same period.

We calculated age-specific breast cancer incidence rates per 100'000 by five-year age groups for four different periods (1975–1979, 1985–1989, 1997–2000, and 2001–2003). Differences between age-specific incidence curves were analyzed by generalized log linear regression analysis [13]. We compared age-specific incidence rates among the 400 patients with information on use of HRT dividing them into four groups of patients: 1) ever used HRT, tumour detected by mammography screening (n = 87), 2) ever used HRT, tumour not detected by mammography screening (n = 141), 3) never used HRT, tumour detected by mammography screening (n = 53), 4) never used HRT, tumour not detected by mammography screening (n = 119).

## Results

**Figure 1:**
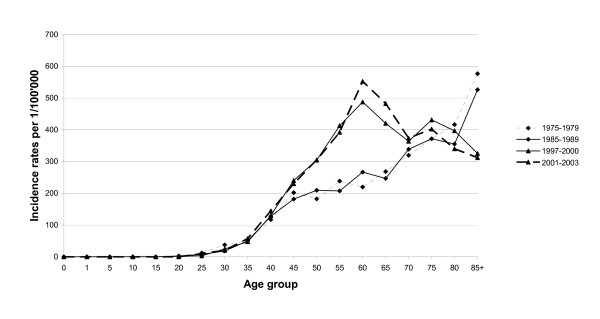
Invasive breast cancer incidence rates by age and period, Geneva cancer registry 1975–2003.

**Figure 2:**
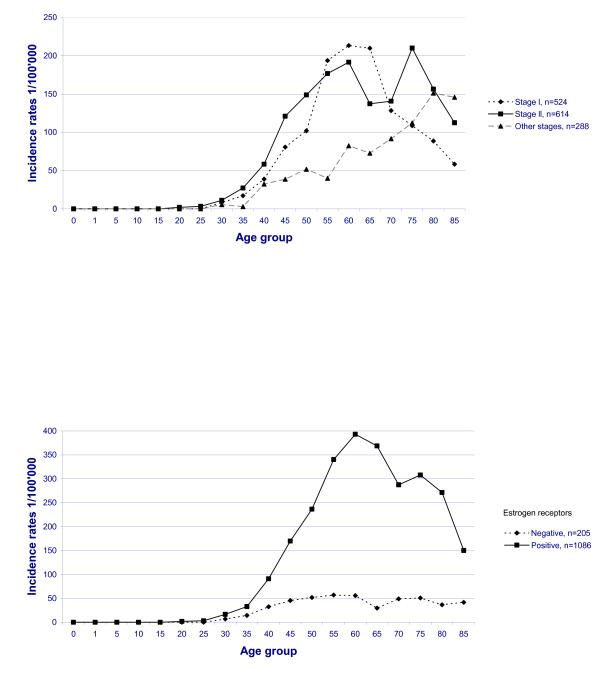
Invasive breast cancer incidence rates according to stage at diagnosis (panel a) and oestrogen receptor status (panel b) 1997–2000. Panel a. Panel b.

Figure 1 shows the change in breast cancer incidence by age. In the periods 1975–1979 and 1985–1989, breast cancer incidence increased with age. Rates among women aged ≥85 years (568/100'000) were at least three times higher than among women aged 50–54 years (182/1000'000). For the period 1997–2000, the age-specific incidence curve was completely different and showed a peak at 60–64 years (488/100'000). In this period, rates among the oldest women (313/100'000) were much lower and comparable to those of women aged 50–54 years (303/100'000). Data from the last available period (2001–2003) confirmed the change in incidence rate by age. The incidence peak among women aged 60–64 years, occurring since 1997, involved mainly early stages (stage I and II) and oestrogen receptor positive tumours (Figure 2). Table I compares the patient and tumour characteristics among women aged 60–64 years before and after the change in age-specific incidence. The proportion of stage I disease rose from 32 to 48% (p < 0.001). Also, the proportion of women of high social class was much higher in the recent years. The proportion of lobular cancer increased sharply: before the change in incidence pattern, lobular cancer represented only 2% of the tumours occurring in women aged 60–64 years, while afterwards, 14% of women aged 60–64 years presented with lobular histology.

**Figure 3:**
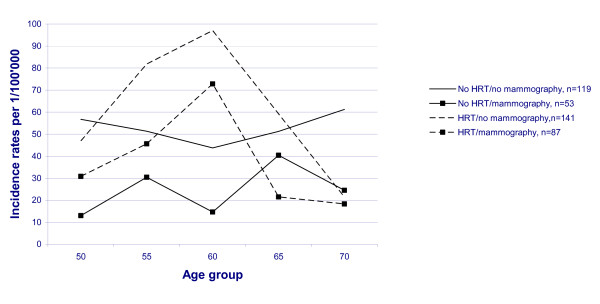
Invasive breast cancer incidence according to ever use of hormone replacement therapy (HRT) and detection by mammography screening, 1997–2000. As information on HRT use was available for 46% of all breast cancer patients, breast cancer incidence rates are approximately half of the overall breast cancer incidence rates during the period 1997–2000.

Figure 3 presents incidence rates by age according to the use of HRT and the method of tumour detection among the 400 patients (aged 50–74 years) with available data on HRT. As information on HRT use was available for 46% of all breast cancer patients, rates were approximately 50% of the overall breast cancer incidence rates for the period 1997–2000. The age-specific incidence curves were significantly different among ever users of HRT compared with never users (P < 0.0001). The incidence peak was observed only among ever users of HRT, regardless whether the tumour was detected by mammography screening or not. For women who never used HRT, there was no incidence peak at 60–64 years.

## Discussion

This study shows a remarkable change in age-specific breast cancer incidence in Geneva. In developed countries, other than Japan, the typical age incidence curve of breast cancer described a progressive increase of risk with age, with a slope down around the menopause age, called the Clemmensen's hook. This typical curve by age is no longer observed in Geneva, where the risk of developing breast cancer does not increase with advancing age anymore. The highest breast cancer risk is now observed among women aged 60–64 years with strongly decreased risks among older women. The incidence peak of women aged 60–64 years was only observed in early stage disease and in oestrogen receptor positive tumours. In the subgroup of women for whom information on HRT use and mammography screening was available, the incidence peak was only present among women who ever used HRT.

The prevalence of screening and HRT use are high in our study population and we had the opportunity to examine the respective effects of each of these factors on the change in age of breast cancer occurrence. However, we realise that our study has several shortcomings. Information on HRT use was available only for approximately 50% of the women diagnosed in the period 1997–2000. Although these women were recruited in the context of a population-based case-control study, they might not be completely representative of all breast cancer patients. We compared them with the other 50–74 years old breast cancer patients recorded at the cancer registry and they appeared to be similar in terms of age, social class, stage distribution and tumour characteristics. Therefore, we believe that selection bias does not explain the fact that the new age distribution is only present among women who used HRT, irrespective of their mammography screening status. In addition, we have no information on the duration of HRT utilisation, on HRT cessation, or on time elapsed since the stop of HRT. It is therefore not possible to evaluate if the incidence peak occurred among current or ex-users of HRT. Also, we were not able to evaluate incidence patterns according to type of HRT.

To our knowledge, only three studies have reported important changes in age-specific breast cancer incidence [14-16]. In Marin Country, Bay area, San Francisco, Prehn et al. observed an increase in breast cancer incidence among women aged 45–64 years for the period 1991–1997, whereas the incidence among older and younger women remained stable [14]. They ruled out screening mammography as the most important reason, because the incidence increase involved also advanced stages and lobular cancers (which are difficult to detect mammographically), and because women aged 65–70 (an age-category also covered by screening) showed no incidence increase. The authors suggested HRT, frequently used by the relatively wealthy, well-educated female population of this area, as a possible explanation for the increasing breast cancer incidence among women aged 45–64. It was in this same region (Bay area) that the increase of endometrial adenocarcinoma, associated with postmenopausal oestrogen use, was particularly high [17]. More recently, Hemminki et al. also reported an important change in age-specific breast cancer incidence in Sweden [16]. They observed a strong incidence increase among women aged 50–69 years, but only a slight decrease for women older than 75 years. As this change in age-specific incidence coincided with the introduction of organised mammography screening programs, the authors attributed this incidence pattern change to mammography screening. Fuglede et al, reported on breast cancer incidence in a population of unscreened women in Denmark from 1973–2002, using data of the nationwide Danish Cancer Registry [15]. They showed that the age-specific incidence rates of breast cancer increased throughout the whole period. In addition, they observed marked changes in the age-specific incidence pattern: between 1973–1981, there was a plateau and change of slope around the age of 46–48, which shifted to 64–66 years in 1994–2002. This was not due to screening as they confined their study to a non-screened population [15].

In our population, we believe that both HRT and, to a lesser extent, mammography screening explain the change in incidence pattern. Mammography screening advances diagnosis by detecting breast cancer at an early pre-clinical stage. This can explain why the change in pattern concerned mainly the early stages. In this study however, we did not observe the typical incidence peak among never users of HRT with screen-detected tumours. In fact, only ever users of HRT showed an incidence peak at 60–64 years, regardless whether the tumour was screen-detected or not.

It has been suggested that HRT particularly increases the risk of oestrogen receptor positive tumours [18]. In our study, the changed incidence pattern involved only women with oestrogen receptor positive tumours, which supports our hypothesis of a potential effect of HRT use on the change in age-specific breast cancer risk.

Recent studies showed that current users of HRT are at increased risk of developing breast cancer [5, 19]. These studies generally involved patients between 50–65 years and the effect of ever use of HRT on breast cancer incidence among elderly women (aged ≥ 75 years) has, to our knowledge, never been examined.

Our results suggest that HRT use might not only increase breast cancer risk among middle-aged women. It may also bring forward the clinical appearance of breast cancer by several years by stimulating the growth of oestrogen sensible lesions and by inhibiting spontaneous slow down of tumours when sex hormone levels drop during menopause. This hypothesis is supported by a previous study, which showed that postmenopausal women using HRT were on average five years younger upon breast cancer diagnosis than never users [20].

Use of HRT could have eliminated the temporary protective effect of menopausal oestrogen depletion.

## Conclusion

The results of this study suggest that HRT may not only increase the breast cancer risk among middle-aged women, but that it may also bring forward the clinical appearance of breast cancer. To get more insight in the complex relationship between age-specific breast cancer incidence, mammography screening and HRT, it could be useful to perform international comparisons of cancer registry data covering populations with different mammography screening policies and different prevalences of HRT use. The change in incidence pattern could have an impact on breast cancer mortality. If the increase is "only" anticipation in age at onset, we can expect that the subsequent mortality will remain stable. However, there is also an anticipation of stage and that could lead to a future lowered mortality. If it is a true increase in risk, then we can expect a subsequent possible increase in mortality.

## Competing interests

The author(s) declare that they have no competing interests.

## Authors' contributions

Christine Bouchardy, Alfredo Morabia, Helena M. Verkooijen, Gérald Fioretta and Peter Schäfer designed the study. Alfredo Morabia, Yves Wespi, Gérald Fioretta and Peter Schäfer were involved in data collection. Christine Bouchardy, Gérald Fioretta and Helena M. Verkooijen performed the statistical data analyses. All authors were involved in the interpretation of the data. All authors read and approved the final manuscript.

## Pre-publication history

The pre-publication history for this paper can be accessed here:



## Figures and Tables

**Table 1:** Patient and tumour characteristics by period of diagnosis among patients aged 60–64 years^1^

Patient and tumour characteristics	1975–1979 1985–1989 N = 218 (%)	1997–2000 2001–2003 N = 389 (%)	P value for X^2 ^of heterogeneity
Social class^2^			
High	8 (6%)	72 (19%)	P < 0.0000
Medium	57 (44%)	203 (52%)	
Low	26 (20%)	53 (14%)	
Unknown	39 (30%)	61 (16%)	
			
Method of detection			
Screening	31 (14%)	199 (51%)	P < 0.0000
Other	187 (86%)	190 (49%)	
			
Stage at diagnosis			
I	69 (32%)	186 (48%)	P < 0.0000
II	95 (44%)	149 (38%)	
III and more	34 (16%)	42 (11%)	
Unknown	20 (09%)	12 (3%)	
			
Oestrogen ^3 ^receptors			
Positive	43 (61%)	311 (80%)	P = 0.002
Negative	18 (25%)	53 (14%)	
Unknown	10 (14%)	25 (6%)	
			
Histological type			
Ductal	173 (79%)	311 (80%)	P < 0.0000
Lobular	4 (2%)	53 (14%)	
Other	41 (19%)	25 (6%)	

1) the age in which the incidence peak was observed after 1997

2) the first period concerned 1985–1989 as information on the woman's last occupation was available only after 1980;

3) the fist period concerned the years 1995–1996 as information on oestrogen receptor status was available only from 1995.
